# Cardiovascular safety of antimuscarinic add‐on therapy in patients with overactive bladder who had a suboptimal response to mirabegron monotherapy: A post hoc analysis from the Japanese MILAI II study

**DOI:** 10.1111/luts.12286

**Published:** 2019-09-30

**Authors:** Takao Katoh, Yasuhiko Igawa, Osamu Yamaguchi, Daisuke Kato, Takuya Hamada, Kentaro Kuroishi

**Affiliations:** ^1^ Cardiovascular Center, Mita Hospital International University of Health and Welfare Tokyo Japan; ^2^ Department of Continence Medicine The University of Tokyo Graduate School of Medicine Tokyo Japan; ^3^ Department of Chemical Biology and Applied Chemistry Nihon University School of Engineering Koriyama Japan; ^4^ Astellas Pharma Inc. Tokyo Japan

**Keywords:** antimuscarinics, cardiovascular, combination therapy, mirabegron, overactive bladder

## Abstract

**Objective:**

This analysis was conducted to investigate the cardiovascular (CV) safety outcomes from the MILAI II study. MILAI II was conducted to evaluate the long‐term safety and efficacy of antimuscarinic add‐on therapy to mirabegron over 52 weeks in patients with overactive bladder (OAB) symptoms.

**Methods:**

MILAI II consisted of a 2‐week screening period (patients received mirabegron 50 mg once daily) plus a 52‐week treatment period (patients were randomized to receive a combination of mirabegron 50 mg/d plus solifenacin 5 mg/d, propiverine 20 mg/d, imidafenacin 0.2 mg/d, or tolterodine 4 mg/d). CV safety was assessed using treatment‐emergent adverse events (TEAEs), vital signs, and 12‐lead electrocardiograms (ECGs). Vital signs and ECG data were evaluated for each patient using worst post‐baseline values reported.

**Results:**

Of 647 patients, 570 (88.1%) were female with a mean age of 65 years. CV history at baseline and CV‐related concomitant medication use throughout the study were balanced between groups. The incidences of overall and drug‐related CV TEAEs were ≤8.1% and ≤6.2%, respectively, for all groups. The most common TEAEs were ECG T wave amplitude decreased, ECG QT prolonged, and ventricular extrasystoles. Overall, 36 TEAEs of interest related to the CV system that were possibly/probably related to treatment were reported with similar incidences for each group. For the worst post‐baseline vital signs and ECGs, no relationships were noted in terms of either timing or treatment group.

**Conclusion:**

A favorable CV safety profile was observed following long‐term combination treatment with mirabegron and an antimuscarinic in patients with OAB symptoms.

## 1 INTRODUCTION

Overactive bladder (OAB) syndrome is a prevalent condition and an estimated 12.4% of the Japanese population who are ≥40 years of age experience symptoms of OAB.^1^ Real‐world studies have indicated that substantially more patients with OAB present with concomitant cardiovascular (CV) comorbidities compared with age‐ and gender‐matched controls.^2^, ^3^ This finding emphasizes the importance of evaluating the CV safety of potential and existing OAB pharmacotherapies.

Antimuscarinic medications currently form the mainstay of pharmacotherapy approaches for treating patients with OAB symptoms.^4^ These medications are believed to act by inhibiting the binding of acetylcholine to the muscarinic receptors M2 and M3 that are found on detrusor smooth muscle cells and other bladder wall components.^5^ A further medication that is used in clinical practice for treating patients with OAB symptoms is the β3‐adrenoreceptor agonist, mirabegron, which may act through various mechanisms, including relaxation of the detrusor muscle by cyclic adenosine monophosphate (cAMP) generation and inhibition of spontaneous contractile activity in the bladder.^6^

As well as in the bladder, preclinical studies have shown that M2 and M3 receptors and the β‐adrenoreceptors (β1, β2, and β3) are also expressed in the CV system.^7^, ^8^ Antagonism of the M2 receptor (which plays a functional role in mediating heart rate)^9^ and the M3 receptor (which mediates vasodilation)^10^ could possibly increase heart rate, prolong the QT interval, and induce potentially fatal ventricular tachyarrhythmias, such as torsade de pointes.^9^ Further investigations have discovered that the β1‐adrenoreceptor mediates increased heart rate and contractility and the β2‐adrenoreceptor mediates vasodilation in the vascular smooth muscle.^11^ The role of the β3‐adrenoreceptor in the physiology of the human CV system is currently less clear, although activation of the β3‐adrenoreceptor is known to induce positive inotropic effects in human atrial tissue and negative inotropic effects in ventricular tissue.^12^

Several clinical studies have also assessed the CV safety of mirabegron or antimuscarinic monotherapy. Antimuscarinic agents appear to have a favorable CV safety profile and CV‐related treatment‐emergent adverse events (TEAEs) are rarely reported.^13^ However, the most commonly reported events of increases in heart rate and QT interval need to be taken into account when prescribing these medications. Furthermore, in a pooled analysis of 12‐week mirabegron monotherapy studies, no trends across treatment groups (placebo, mirabegron, tolterodine) were observed in the frequencies of abnormal electrocardiogram (ECG) findings.^14^

Although mirabegron and antimuscarinics are effective monotherapies for patients with OAB symptoms, poor responses to treatment have been noted.^15^, ^16^ These patients may achieve an improved outcome if they subsequently receive combination therapy involving mirabegron plus an antimuscarinic. However, there is a concern that combining mirabegron with antimuscarinics may result in synergistic effects on the CV system.

Several international phase II‐IV clinical trials (Symphony,^17^ BESIDE,^18^ MILAI,^19^ SYNERGY,^20^ and SYNERGY II^21^) have been conducted to assess the efficacy and safety of mirabegron with the antimuscarinic, solifenacin. The combination of mirabegron with solifenacin resulted in improved efficacy over the monotherapies^17^, ^18^, ^20^, ^21^ and no synergistic CV effects were reported in subanalyses from the BESIDE and SYNERGY trials.^22^, ^23^ However, both of these trials were conducted in Western countries and therefore it is important to assess the CV safety of mirabegron and antimuscarinic combination therapy in Asian patients.

MILAI II was a 52‐week study involving 649 Japanese patients with residual OAB symptoms.^24^ The results of the study showed that antimuscarinic add‐on therapy (solifenacin, propiverine, imidafenacin, or tolterodine) was well tolerated and effective following ≥6 weeks of initial treatment with mirabegron. Herein, we report the findings of a post hoc analysis that evaluated the CV safety outcomes from the MILAI II study. In addition, using the vital sign data, we examined whether there are any timing factors that need to be taken into account after commencing combination therapy.

## 2 METHODS

The overall methodology has been previously published.^24^ In summary, MILAI II (http://clinicaltrials.gov: NCT02294396) was a multicenter (60 sites in Japan), randomized, open‐label, phase IV study which was conducted from October 2014 to September 2016. Safety was the primary objective of the study and efficacy was the secondary objective.

The Declaration of Helsinki and International Council for Harmonisation guidelines were adhered to throughout the study. All participants provided informed consent and the institutional review board for each site approved the protocol.

Eligible patients had to be ≥20 years of age, been receiving mirabegron treatment (50 mg) for ≥6 weeks, and have residual OAB symptoms (total OAB symptom score [OABSS] ≥3 points, question 3 OABSS ≥2 points). The study consisted of a 2‐week screening period, during which eligible patients received oral mirabegron 50 mg once daily after breakfast, plus a 52‐week treatment period. After completion of the screening period, patients were randomized to receive a combination of mirabegron 50 mg/d plus either solifenacin 5 mg/d, propiverine 20 mg/d, imidafenacin 0.2 mg/d, or extended‐release tolterodine 4 mg/d (1:1:1:1 ratio). At week 8, the dose of solifenacin, propiverine, or imidafenacin could be doubled. However, if a TEAE developed after the dose increase, the investigator could reduce the dose to its original level.

CV‐related concomitant medications were recorded throughout the study. CV safety was assessed using TEAEs, vital signs, and 12‐lead ECGs (including QT interval corrected for heart rate by Fridericia's formula [QTcF] measurements). The Japanese version of the Medical Dictionary for Regulatory Activities (MedDRA/J, version 17.0) was used to cross‐reference the reported terms from the study physicians to the relevant system organ classes and preferred terms. At rest vital sign and ECG data were examined in terms of worst post‐baseline values reported for each patient in the clinic with only increases from baseline included in the analyses. CV history, CV safety, and demographic data were evaluated using the safety analysis set (SAF), which was defined as patients who had received ≥1 dose of study drug.

## 3 RESULTS

### 3.1 Patient characteristics

Out of 649 randomized patients, 647 were included in the SAF; one patient from the mirabegron and solifenacin group did not complete a second informed consent form, and one patient from the mirabegron and tolterodine group did not take any study medication.^24^ Most of the patients enrolled were female (570 [88.1%] patients), with a mean age of 65 years. All four treatment groups were generally similar regarding patient demographics, baseline characteristics, and CV history (Table 1). Similar proportions of patients from each group received a CV‐related concomitant medication during the study (mirabegron and solifenacin group: 58 [34.9%] patients, mirabegron and propiverine group: 51 [31.7%] patients, mirabegron and imidafenacin group: 56 [34.8%] patients, mirabegron and tolterodine group: 51 [32.1%] patients).

**Table 1:** Patient demographics and baseline characteristics

Variable	MIRA + SOLI (n = 166)	MIRA + PRO (n = 161)	MIRA + IMI (n = 161)	MIRA + TOL (n = 159)	Total (n = 647)
Sex, n (%)					
Male	20 (12.0)	17 (10.6)	15 (9.3)	25 (15.7)	77 (11.9)
Female	146 (88.0)	144 (89.4)	146 (90.7)	134 (84.3)	570 (88.1)
Age in y					
Mean (SD, range)	64.6 (9.4, 45–89)	64.0 (9.3, 42–82)	65.7 (8.7, 47–85)	65.7 (10.0, 40–85)	65.0 (9.4, 40–89)
Age group, n (%)					
<65 y	86 (51.8)	82 (50.9)	65 (40.4)	65 (40.9)	298 (46.1)
≥65 y	80 (48.2)	79 (49.1)	96 (59.6)	94 (59.1)	349 (53.9)
BMI in kg/m^2^					
Mean (SD, range)	23.32 (3.99, 14.8–39.0)	23.14 (3.90, 16.0–34.3)	22.87 (3.47, 15.6–34.6)	23.21 (3.85, 16.4–44.3)	23.13 (3.81, 14.8–44.3)
Duration of OAB in mo					
Mean (SD) [n]	69.3 (68.2) [162]	78.8 (88.9) [158]	83.3 (94.2) [156]	77.9 (85.8) [155]	77.2 (84.7) [631]
Median (range)	49.0 (1–334)	53.0 (1–602)	59.0 (1–545)	55.0 (1–565)	55.0 (1–602)
Previous CV history, n (%)					
Overall	66 (39.8)	63 (39.1)	71 (44.1)	64 (40.3)	264 (40.8)
Vascular disorders	65 (39.2)	61 (37.9)	70 (43.5)	64 (40.3)	260 (40.2)
Hypertension	65 (39.2)	57 (35.4)	67 (41.6)	61 (38.4)	250 (38.6)
Peripheral vascular disorder	0	3 (1.9)	0	1 (0.6)	4 (0.6)
Varicose vein	0	0	1 (0.6)	2 (1.3)	3 (0.5)
Peripheral arterial occlusive disease	0	0	2 (1.2)	0	2 (0.3)
Peripheral circulatory failure	0	1 (0.6)	1 (0.6)	0	2 (0.3)
Aortic aneurysm	1 (0.6)	0	0	0	1 (0.2)
Deep vein thrombosis	0	0	1 (0.6)	0	1 (0.2)
Essential hypertension	0	0	0	1 (0.6)	1 (0.2)
Hypotension	0	1 (0.6)	0	0	1 (0.2)
Lymphedema	0	0	1 (0.6)	0	1 (0.2)
Peripheral artery aneurysm	0	1 (0.6)	0	0	1 (0.2)
Peripheral coldness	0	0	1 (0.6)	0	1 (0.2)
Venous thrombosis	0	0	0	1 (0.6)	1 (0.2)
Cardiac disorders	6 (3.6)	2 (1.2)	3 (1.9)	1 (0.6)	12 (1.9)
Bundle branch block right	2 (1.2)	1 (0.6)	0	0	3 (0.5)
Angina pectoris	1 (0.6)	0	0	0	1 (0.2)
Aortic valve stenosis	0	1 (0.6)	0	0	1 (0.2)
Arteriosclerosis coronary artery	0	0	1 (0.6)	0	1 (0.2)
Atrioventricular block first degree	1 (0.6)	0	0	0	1 (0.2)
Mitral valve prolapse	1 (0.6)	0	0	0	1 (0.2)
Palpitations	0	0	1 (0.6)	0	1 (0.2)
Supraventricular extrasystoles	0	0	0	1 (0.6)	1 (0.2)
Supraventricular tachycardia	1 (0.6)	0	0	0	1 (0.2)
Tachycardia	1 (0.6)	0	0	0	1 (0.2)
Ventricular extrasystoles	0	0	1 (0.6)	0	1 (0.2)
Investigations	1 (0.6)	2 (1.2)	0	0	3 (0.5)
ECG QT prolonged	1 (0.6)	2 (1.2)	0	0	3 (0.5)
ECG PR prolongation	0	1 (0.6)	0	0	1 (0.2)
ECG ST segment depression	0	1 (0.6)	0	0	1 (0.2)
ECG T wave amplitude decreased	0	1 (0.6)	0	0	1 (0.2)
ECG T wave inversion	0	1 (0.6)	0	0	1 (0.2)
Vital signs					
Mean SBP in mm Hg (SD, range)	126.6 (15.7, 86–170)	124.6 (15.3, 86–170)	124.5 (17.1, 84–178)	126.4 (16.9, 82–174)	NC
Mean DBP in mm Hg (SD, range)	77.6 (9.7, 54–105)	76.6 (11.1, 50–104)	75.7 (10.9, 33–105)	75.5 (11.0, 40–100)	NC
Mean pulse rate in bpm (SD, range)	73.7 (9.5, 52–101)	74.1 (9.5, 52–102)	73.1 (8.3, 52–99)	73.4 (8.6, 55–97)	NC
ECG parameters					
Mean QTcF in ms (SD) [n]	418.5 (17.4) [164]	419.2 (16.9) [161]	416.4 (17.3) [160]	415.4 (15.6) [158]	NC

*Note*: Data shown for the safety analysis set (patients who received ≥1 dose of study drug).

*Note*: The sex, age, duration of OAB, and ECG parameter data have been previously published.^24^

Abbreviations: BMI, body mass index; CV, cardiovascular; DBP, diastolic blood pressure; ECG, electrocardiogram; IMI, imidafenacin; MIRA, mirabegron; NC, not calculated; OAB, overactive bladder; PRO, propiverine; QTcF, QT interval corrected for heart rate by Fridericia's formula; SBP, systolic blood pressure; SD, standard deviation; SOLI, solifenacin; TOL, tolterodine.

### 3.2 CV events

The overall safety and efficacy results from the MILAI II study have been previously presented.^24^ In total, 519 (80.2%) patients experienced ≥1 TEAE and 303 (46.8%) patients experienced ≥1 drug‐related TEAE. In addition, 28 (4.3%) patients reported ≥1 serious TEAE. Two serious TEAEs were considered to be possibly drug‐related by the investigator, one of which was a CV‐related event in a patient from the mirabegron and propiverine group who experienced atrial fibrillation; this event resolved 10 days after treatment withdrawal.

**Figure 1:**
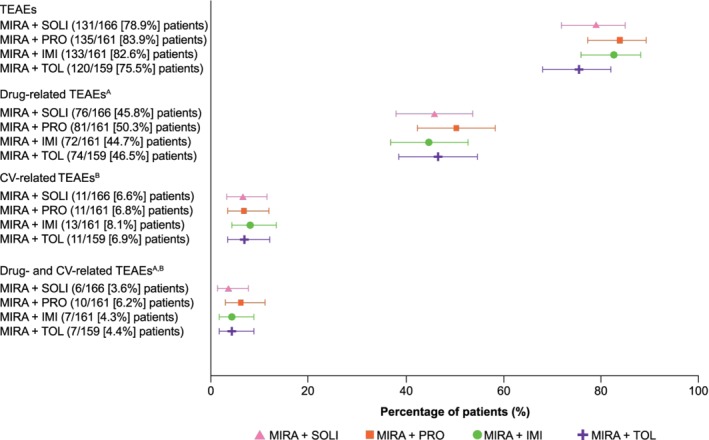
Dot and whiskers plot of CV‐related TEAEs. Data shown for the safety analysis set (patients who received ≥1 dose of study drug). The dots represent the percentage of patients from each group who experienced a TEAE from each particular category and the whiskers represent the corresponding 95% confidence intervals. The TEAE and drug‐related TEAE data have been previously published.^24^ A, A reasonable possibility that the event may have been caused by the study drug, as assessed by the investigator. If the relationship was missing then it was considered to be drug‐related. B, Includes serious adverse events evaluated by the investigator. CV, cardiovascular; IMI, imidafenacin; MIRA, mirabegron; PRO, propiverine; SOLI, solifenacin; TEAE, treatment‐emergent adverse event; TOL, tolterodine

The incidence of CV‐related TEAEs was similar between groups (Figure 1 and Table 2). The overall and drug‐related incidence rates were ≤8.1% and ≤6.2%, respectively, for all treatment groups. The most common overall CV‐related TEAEs were ECG T wave amplitude decreased (10 [1.5%] patients), ECG QT prolonged (nine [1.4%] patients), and ventricular extrasystoles (seven [1.1%] patients). The most common drug‐related TEAEs were ECG T wave amplitude decreased (eight [1.2%] patients), ECG QT prolonged (eight [1.2%] patients), and supraventricular extrasystoles (four [0.6%] patients). The overall and drug‐related incidences of ECG QT prolonged were slightly higher in the mirabegron and imidafenacin group compared with the other three groups.

**Table 2:** TEAEs of interest related to the CV system

	TEAE, n (%)				Drug‐related TEAE, n (%)			
System organ class/preferred term	MIRA + SOLI (n = 166)	MIRA + PRO (n = 161)	MIRA + IMI (n = 161)	MIRA + TOL (n = 159)	MIRA + SOLI (n = 166)	MIRA + PRO (n = 161)	MIRA + IMI (n = 161)	MIRA + TOL (n = 159)
Overall	11 (6.6)	11 (6.8)	13 (8.1)	11 (6.9)	6 (3.6)	10 (6.2)	7 (4.3)	7 (4.4)
Investigations	5 (3.0)	5 (3.1)	6 (3.7)	5 (3.1)	3 (1.8)	5 (3.1)	5 (3.1)	4 (2.5)
ECG T wave amplitude decreased	3 (1.8)	3 (1.9)	1 (0.6)	3 (1.9)	2 (1.2)	3 (1.9)	1 (0.6)	2 (1.3)
ECG QT prolonged	1 (0.6)	2 (1.2)	5 (3.1)	1 (0.6)	1 (0.6)	2 (1.2)	4 (2.5)	1 (0.6)
ECG T wave inversion	0	2 (1.2)	1 (0.6)	0	0	1 (0.6)	1 (0.6)	0
Blood pressure decreased	1 (0.6)	0	0	0	0	0	0	0
ECG change	0	0	0	1 (0.6)	0	0	0	1 (0.6)
ECG U‐wave abnormality	1 (0.6)	0	0	0	0	0	0	0
Cardiac disorders	4 (2.4)	6 (3.7)	4 (2.5)	4 (2.5)	2 (1.2)	5 (3.1)	1 (0.6)	4 (2.5)
Ventricular extrasystoles	3 (1.8)	1 (0.6)	1 (0.6)	2 (1.3)	1 (0.6)	0	0	2 (1.3)
Supraventricular extrasystoles	0	2 (1.2)	3 (1.9)	1 (0.6)	0	2 (1.2)	1 (0.6)	1 (0.6)
Atrial fibrillation	1 (0.6)	1 (0.6)	0	0	1 (0.6)	1 (0.6)	0	0
Bundle branch block right	0	0	0	2 (1.3)	0	0	0	1 (0.6)
Sinus tachycardia	0	1 (0.6)	0	1 (0.6)	0	1 (0.6)	0	1 (0.6)
Arrhythmia supraventricular	0	0	1 (0.6)	0	0	0	0	0
Atrial tachycardia	0	0	1 (0.6)	0	0	0	1 (0.6)	0
Bundle branch block left	0	1 (0.6)	0	0	0	1 (0.6)	0	0
Vascular disorders	2 (1.2)	0	3 (1.9)	3 (1.9)	1 (0.6)	0	1 (0.6)	0
Hypertension	2 (1.2)	0	2 (1.2)	1 (0.6)	1 (0.6)	0	1 (0.6)	0
Aortic aneurysm	0	0	1 (0.6)	0	0	0	0	0
Deep vein thrombosis	0	0	0	1 (0.6)	0	0	0	0
Microscopic polyangiitis	0	0	0	1 (0.6)	0	0	0	0

*Note*: Data shown for the safety analysis set (patients who received ≥1 dose of study drug). The preferred terms of interest were defined as aortic aneurysm, arrhythmia supraventricular, atrial fibrillation, atrial tachycardia, blood pressure ambulatory decreased, blood pressure decreased, blood pressure increased, bundle branch block left, bundle branch block right, deep vein thrombosis, ECG change, ECG PR prolongation, ECG QT prolonged, ECG T wave amplitude decreased, ECG T wave inversion, ECG U‐wave abnormality, hypertension, microscopic polyangiitis, sinus tachycardia, supraventricular extrasystoles, and ventricular extrasystoles.

Abbreviations: CV, cardiovascular; ECG, electrocardiogram; IMI, imidafenacin; MIRA, mirabegron; PRO, propiverine; SOLI, solifenacin; TEAE, treatment‐emergent adverse event; TOL, tolterodine.

In total, 36 TEAEs of interest related to the CV system that were possibly or probably related to mirabegron and/or the combination antimuscarinic drug were reported during the MILAI II study (Table 3). Of these, seven, 11, nine, and nine TEAEs were reported by the patients from the mirabegron and solifenacin, mirabegron and propiverine, mirabegron and imidafenacin, and mirabegron and tolterodine groups, respectively. The events occurred between 21 and 364 days after the start of combination treatment and no discernible differences in time of onset were noted between groups. The majority of the TEAEs were mild in severity (34 [94.4%] events) and the rest were moderate (two [5.6%] events). In total, 23 (63.9%) TEAEs had resolved or were resolving by the end of the study. No changes in mirabegron and antimuscarinic drug dose were required for the majority of the TEAEs (28 [77.8%] events) and drug treatment was withdrawn in the other cases (eight [22.2%] events).

**Table 3:** Listing of drug‐related TEAEs of interest related to the CV system

Age in years	Sex	System organ class/preferred term/reported term^a^	Onset day/end day	Last dose day	Serious (reason)^b^/severity	Outcome/treatment required	Relationship to MIRA/action taken	Relationship to antimuscarinic/action taken	CV history
MIRA + SOLI									
60	F	Investigations/ECG T wave amplitude decreased/T wave flattening	364/UNK	364	No/mild	Not resolved/none	Possible/dose not changed	Possible/dose not changed	
80	F	Vascular disorders/ hypertension/ deterioration of hypertension	285/364	364	No/mild	Resolved/none	Possible/dose not changed	Possible/dose not changed	Hypertension
53	F	Investigations/ECG T wave amplitude decreased/T wave flattening	196/278	362	No/mild	Resolved/none	Possible/dose not changed	Possible/dose not changed	
68	F	Cardiac disorders/ ventricular extrasystoles/frequent premature ventricular complex	364/UNK	364	No/mild	Not resolved/none	Possible/dose not changed	Possible/dose not changed	Hypertension
64	F	Investigations/ECG QT prolonged/QT interval prolonged	115/274	361	No/mild	Resolved/none	Possible/dose not changed	Possible/dose not changed	
Same patient as above		Investigations/ECG QT prolonged/QTc prolonged	361/UNK	361	No/mild	Not resolved/none	Possible/dose not changed	Possible/dose not changed	
83	F	Cardiac disorders/atrial fibrillation/atrial fibrillation	58/UNK	58	No/mild	Not resolved/none	Possible/drug withdrawn	Possible/drug withdrawn	Hypertension
MIRA + PRO									
66	F	Cardiac disorders/atrial fibrillation/atrial fibrillation with tachycardia	49/UNK	49	Yes (OMI)/mild	Not resolved/drug treatment	Possible/drug withdrawn	Possible/drug withdrawn	
76	M	Cardiac disorders/ supraventricular extrasystoles/ supraventricular extrasystoles	58/UNK	200	No/mild	Not resolved/none	Possible/dose not changed	Possible/dose not changed	Hypertension
63	F	Investigations/ECG T wave amplitude decreased/T wave flattening	56/84	84	No/mild	Resolved/none	Possible/dose not changed	Possible/dose not changed	Hypertension
Same patient as above		Investigations/ECG T wave inversion/ negative T wave	56/84	84	No/mild	Resolved/none	Possible/dose not changed	Possible/dose not changed	
52	F	Cardiac disorders/sinus tachycardia/sinus tachycardia	56/UNK	362	No/mild	Not resolved/none	Possible/dose not changed	Possible/dose not changed	Hypertension
59	F	Cardiac disorders/bundle branch block left/ complete left bundle branch block	91/106	105	No/moderate	Resolved/none	Possible/drug withdrawn	Possible/drug withdrawn	Hypertension
74	F	Cardiac disorders/ supraventricular extrasystoles/ premature atrial contraction	32/51	368	No/mild	Resolved/none	Possible/dose not changed	Possible/dose not changed	
74	F	Investigations/ECG QT prolonged/ deterioration of QT prolonged	105/UNK	112	No/mild	Not resolved/none	Possible/drug withdrawn	Possible/drug withdrawn	Complete right bundle branch block, PR prolonged, QT interval prolonged
57	F	Investigations/ECG T wave amplitude decreased/T wave flattening	194/278	369	No/mild	Resolved/none	Possible/dose not changed	Possible/dose not changed	
79	F	Investigations/ECG T wave amplitude decreased/enhanced T wave flattening	28/43	43	No/mild	Resolved/none	Possible/drug withdrawn	Possible/drug withdrawn	T wave flattening, negative T wave, mild ST depressed, mild QTc prolonged
60	F	Investigations/ECG QT prolonged/QT interval prolonged	119/UNK	364	No/moderate	Resolving/none	Possible/dose not changed	Possible/dose not changed	
MIRA + IMI									
53	F	Investigations/ECG QT prolonged/QTc prolonged	61/90	364	No/mild	Resolved/none	Possible/dose not changed	Possible/dose not changed	
67	F	Investigations/ECG T wave inversion/ negative T wave	49/UNK	357	No/mild	Not resolved/none	Probable/dose not changed	Probable/dose not changed	
Same patient as above		Investigations/ECG QT prolonged/mild QT prolonged	49/119	357	No/mild	Resolved/none	Possible/dose not changed	Possible/dose not changed	
53	F	Investigations/ECG QT prolonged/QT interval prolonged	35/357	357	No/mild	Resolved/none	Probable/dose not changed	Possible/dose not changed	
52	F	Vascular disorders/ hypertension/ deterioration of hypertension	270/364	364	No/mild	Resolved/drug treatment	Possible/dose not changed	Possible/dose not changed	Hypertension
83	F	Cardiac disorders/atrial tachycardia/ectopic atrial tachycardia	28/UNK	45	No/mild	Not resolved/none	Possible/drug withdrawn	Possible/drug withdrawn	
Same patient as above		Cardiac disorders/ supraventricular extrasystoles/ premature atrial contraction	28/45	45	No/mild	Resolved/none	Possible/drug withdrawn	Possible/drug withdrawn	
65	F	Investigations/ECG T wave amplitude decreased/T wave flattening	112/287	266	No/mild	Resolved/none	Possible/dose not changed	Possible/dose not changed	Hypertension
57	F	Investigations/ECG QT prolonged/QTcF prolonged	113/195	365	No/mild	Resolved/none	Possible/dose not changed	Possible/dose not changed	
MIRA + TOL									
61	F	Investigations/ECG T wave amplitude decreased/T wave flattening	364/UNK	364	No/mild	Not resolved/none	Possible/dose not changed	Possible/dose not changed	Hypertension
70	F	Investigations/ECG change/ECG change	21/UNK	32	No/mild	Not resolved/none	Possible/drug withdrawn	Possible/drug withdrawn	
63	F	Cardiac disorders/ ventricular extrasystoles/ premature ventricular complex	28/56	259	No/mild	Resolved/none	Possible/dose not changed	Possible/dose not changed	White coat hypertension
Same patient as above		Cardiac disorders/sinus tachycardia/sinus tachycardia	28/112	259	No/mild	Resolved/none	Possible/dose not changed	Possible/dose not changed	
71	F	Investigations/ECG T wave amplitude decreased/T wave flattening	105/196	364	No/mild	Resolved/none	Possible/dose not changed	Possible/dose not changed	Hypertension
75	F	Cardiac disorders/bundle branch block right/ complete right bundle branch block	28/49	114	No/mild	Resolved/none	Not related/dose not changed	Possible/dose not changed	
Same patient as above		Investigations/ECG QT prolonged/mild QT prolonged	28/49	114	No/mild	Resolved/none	Not related/dose not changed	Possible/dose not changed	
81	F	Cardiac disorders/ supraventricular extrasystoles/frequent premature atrial contraction	49/UNK	49	No/mild	Not resolved/none	Not related/dose not changed	Possible/dose not changed	Hypertension
71	M	Cardiac disorders/ ventricular extrasystoles/ premature ventricular complex	28/56	357	No/mild	Resolved/none	Possible/dose not changed	Possible/dose not changed	Hypertension

*Note*: The preferred terms of interest were defined as aortic aneurysm, arrhythmia supraventricular, atrial fibrillation, atrial tachycardia, blood pressure ambulatory decreased, blood pressure decreased, blood pressure increased, bundle branch block left, bundle branch block right, deep vein thrombosis, ECG change, ECG PR prolongation, ECG QT prolonged, ECG T wave amplitude decreased, ECG T wave inversion, ECG U‐wave abnormality, hypertension, microscopic polyangiitis, sinus tachycardia, supraventricular extrasystoles, and ventricular extrasystoles.

Abbreviations: CV, cardiovascular; ECG, electrocardiogram; F, female; IMI, imidafenacin; M, male; MIRA, mirabegron; PRO, propiverine; QTcF, QT interval corrected for heart rate by Fridericia's formula; SOLI, solifenacin; TEAE, treatment‐emergent adverse event; TOL, tolterodine; UNK, unknown.

a The preferred terms and the reported terms have both been included to aid understanding.

b Serious TEAEs were identified by the investigator. Reason for seriousness: CA, congenital anomaly; D, death; LT, life‐threatening; OMI, other medical importance; PSDI, persistent or significant disability/incapacity; RPH, requires or prolongs hospitalization.

**Figure 2:**
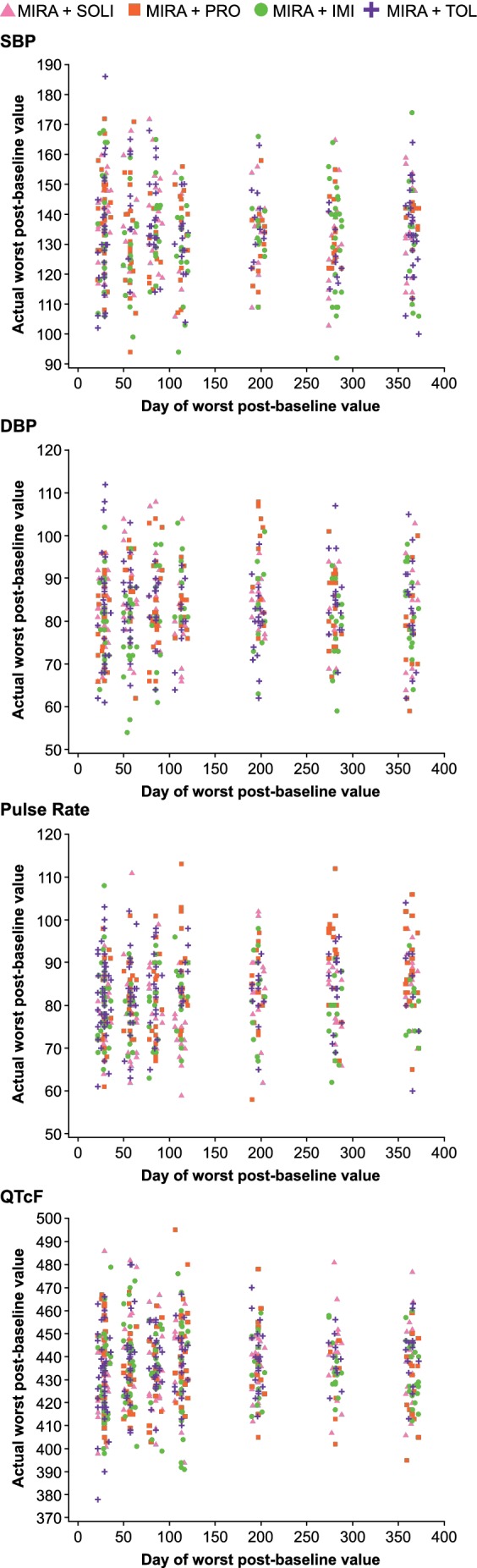
Scatter plot of day of worst cases in vital signs and QTcF at clinical site. Data shown for the safety analysis set (patients who received ≥1 dose of study drug). The data are displayed according to the day when each patient experienced the largest change in each parameter (only increases from baseline were included in the analyses). DBP, diastolic blood pressure; IMI, imidafenacin; MIRA, mirabegron; PRO, propiverine; QTcF, QT interval corrected for heart rate by Fridericia's formula; SBP, systolic blood pressure; SOLI, solifenacin; TOL, tolterodine

The scatter plots for the worst post‐baseline values in systolic blood pressure (SBP), diastolic blood pressure (DBP), pulse rate, and QTcF are shown in Figure 2. For each parameter, the data are displayed according to the day when each patient experienced the largest increase from baseline. No obvious differences between groups were apparent for any of the parameters. Furthermore, no relationships were noted between the increases observed and the time of the highest increase for each patient and the values obtained were evenly dispersed throughout the observation period. The SBP measurements varied between 92 mm Hg (mirabegron and imidafenacin group) and 186 mm Hg (mirabegron and tolterodine group), the DBP measurements varied between 54 mm Hg (mirabegron and imidafenacin group) and 112 mm Hg (mirabegron and tolterodine group), the pulse rate measurements varied between 58 and 113 bpm (both mirabegron and propiverine group), and the QTcF measurements varied between 378 ms (mirabegron and tolterodine group) and 495 ms (mirabegron and propiverine group).

## 4 DISCUSSION

This is the first long‐term study to investigate the CV safety of antimuscarinic add‐on therapy in patients with OAB symptoms following initial mirabegron treatment. This study showed that the incidence of CV‐related TEAEs was below 9% and similar rates were observed in all treatment groups.

In support of the findings presented herein, previous combination studies have indicated that mirabegron in combination with solifenacin has a favorable CV safety profile and no synergistic CV effects are observed with the combination.^22^, ^23^ In the CV subanalyses from the BESIDE and SYNERGY trials, lower overall incidences of CV‐related TEAEs were reported with mirabegron and solifenacin combination therapy (BESIDE: 0%‐1.7% [depending on the TEAE of interest], SYNERGY: 2.8%) compared with the present study (6.6%). The potential reasons for this disparity include that patients received combination therapy for 52 weeks in MILAI II, whereas patients who participated in the BESIDE and SYNERGY trials only received combination therapy for 12 weeks. In addition, different preferred terms of interest were analyzed in this subanalysis compared with the previous studies involving combination treatment. In support of this latter point, similar incidences of CV TEAEs were observed in the MILAI II, BESIDE, and SYNERGY studies when identical preferred terms were included for analysis. For example, hypertension was reported by between 1.1% and 1.3% of the patients who received mirabegron 50 mg and solifenacin 5 mg in combination in all three studies. In the present study, only one drug‐related serious CV‐related TEAE of atrial fibrillation was reported. In support of this finding, none of the seven serious CV‐related TEAEs reported in the BESIDE study were judged to be related to study treatment.^22^

The results of this study add to the wealth of CV‐related data that have been amassed during the clinical development of mirabegron. For example, low incidences of CV‐related events have been noted in phase III trials with mirabegron monotherapy,^25^ although small, statistically significant increases in pulse rate of approximately 1 bpm have also been observed.^14^, ^26^, ^27^ However, these increases in pulse rate were reversed once treatment was discontinued^27^ and were considered to be clinically acceptable. An analysis of pooled data from mirabegron clinical trials that included almost 13 400 patients who had received ≥1 dose of mirabegron, comparator antimuscarinics (solifenacin or tolterodine), or placebo found no evidence of increased CV risk for mirabegron or antimuscarinics in comparison with placebo.^28^ The authors of this analysis concluded that the CV‐related TEAEs reported appeared to be related to patients' pre‐existing conditions, rather than OAB treatment. Importantly, there was also no evidence that OAB treatment or associated blood pressure increases augmented the risk of CV‐related TEAEs. Additional studies have also investigated the effect of mirabegron treatment on ECG parameters. In a thorough QT study involving 352 healthy subjects, mirabegron was associated with QT interval prolongation at the supratherapeutic dose of 200 mg in women.^29^ However, at the therapeutic daily dose of 50 mg, the use of mirabegron was not associated with significant prolongation of QT/QTc interval in either sex. Despite the above findings, Japanese authorities currently recommend caution in administering mirabegron to patients with known CV disease.^30^

Cardiac risk factors may be included as exclusion criteria for clinical trials or may make patients less likely to be included in these studies following assessment by the investigator. For example, trials of mirabegron typically exclude patients with a prolonged QT interval or those taking drugs that are likely to prolong the QT interval.^25^ Specific exclusion criteria in the MILAI II study included long QT syndrome, an abnormal ECG, or a QTcF of ≥450 ms.^24^ One of the limitations of this study is therefore that the incidence of severe CV disease was potentially lower than that in a real‐world population. However, an observational, post‐marketing study has been conducted to investigate the CV safety of mirabegron in 236 Japanese patients with OAB and concomitant CV disease (mean age: 74.5 years).^31^ In the study, 3.4% of the patient population were taking other medication that could cause QT prolongation and 7.5% had a baseline QTcF >450 ms. Although mean heart rate increased by 1.24 bpm after 4 weeks of treatment, this change was not considered to be clinically significant. Furthermore, no significant changes in PR, QRS (ventricular depolarization), or QTcF intervals were noted during the investigation.

In addition, the present study examined vital sign and ECG fluctuations throughout the treatment period. Previous clinical studies have examined average vital sign results over time,^32^, ^33^, ^34^ although we believe that this is the first mirabegron study to examine the most variable values obtained for each patient. The results of the present study showed that no relationships were apparent in the increases in vital signs or QTcF values observed and either the timing of the increase or the treatment administered.

In conclusion, the results of this subanalysis demonstrate the favorable CV safety profile of long‐term treatment with mirabegron in combination with the antimuscarinics, solifenacin, propiverine, imidafenacin, or tolterodine. Physicians can therefore be reassured about the CV safety of these combination therapies when treating patients with OAB within their clinical practice.

## CONFLICTS OF INTEREST

Takao Katoh has received non‐financial support and consultancy fees from Astellas Pharma and consultancy fees from Sumitomo Dainippon Pharma, Ono, and Kissei. Yasuhiko Igawa has received non‐financial support, medical writing assistance, research grants, and consultancy, lectureship, and advisory board member fees from Astellas Pharma; research grants and consultancy and lectureship fees from Pfizer, Kissei, and Nippon Shinyaku; research grants and lectureship fees from Kyorin, Daiichi Sankyo, Ono, and Taiho; and research grants from RaQualia. Osamu Yamaguchi has received non‐financial support, medical writing assistance, and consultancy, lectureship, and advisory board member fees from Astellas Pharma; grants and consultancy and lectureship fees from Hisamitsu; lectureship fees from Pfizer; consultancy fees from Taiho; and grants from Asahi Kasei. Daisuke Kato, Takuya Hamada, and Kentaro Kuroishi are all employees of Astellas Pharma Inc.

## DATA SHARING STATEMENT

Access to anonymized individual participant level data will not be provided for this trial as it meets one or more of the exceptions described on http://www.clinicalstudydatarequest.com under “Sponsor Specific Details for Astellas.”
